# Penoscrotal Elephantiasis nostras verrucosa: A case report and literature review

**DOI:** 10.1016/j.ijscr.2019.10.070

**Published:** 2019-11-02

**Authors:** Yasmin Moussa, Mohamad Moussa, Mohamed Abou Chakra

**Affiliations:** a Clinic of Dermatology, Dr. Brinkmann, Schult & Samimi-Fard. Barbarastraße 15, 45964 Gladbeck, Germany; b Zahraa Hospital, University Medical Center, Lebanese University, Beirut, Lebanon; c Faculty of Medical Sciences, Department of Urology, Lebanese University, Beirut, Lebanon

**Keywords:** Elephantiasis nostras verrucosa, Penoscrotal, Lymphedema, Acitretin, Case report

## Abstract

**Background:**

Elephantiasis Nostras Verrucosa (ENV) is a rare clinical condition associated with chronic non-filarial lymphedema caused by bacterial or non-infectious lymphatic obstruction.

**Case description:**

We reported an unusual case of isolated peno-scrotal ENV of a 67-year-old man with a history of Gastrointestinal Stromal Tumors (GIST) of the stomach. Our patient presented for progressive painless lymphedema of the penis and scrotum since 8 years ago before the diagnosis of GIST tumors, associated with skin changes consistent of superimposed hyperkeratotic papulonodules with a verrucose or cobblestone-like appearance. The patient refused surgical excision. He was treated with oral Acitretin 30 mg/day for 4 weeks where a minimal improvement was noted. Acitretin was stopped due to an increase in liver enzymes. Conservative treatment was applied to decrease lymphostasis.

**Discussion:**

ENV is characterized by lymphedema and skin changes consisting of hyperkeratotic, verrucous and papillomatous lesions. It is most commonly caused by bacterial infection, trauma, neoplasia and obesity. The diagnosis of ENV is achieved clinically by history and typical skin changes. Imagery and skin biopsy are used to differentiate ENV from other diseases.

Management of ENV remains challenging. Strategies to reduce lymph stasis include lymphatic massages, compressive dressings are often inadequate. Surgical debridement may be considered in cases where there is no improvement depsite medical therapy. Oral retinoids can be used if tolerated with variable results.

**Conclusion:**

ENV is a rare disorder that results from chronic obstructive lymphedema. There is no standard therapy but a variety of medical and surgical treatment options have been reported.

## Highlights

• ENV is a rare disorder that results from chronic obstructive lymphedema.
• History and physical examination are sufficient to diagnose ENV.
• Etiologies of lymphatic obstruction can be a bacterial infection, malignancy, and trauma.
• Treatments for ENV include conservative measures to reduce lymphostasis.
• Surgical excision is performed in cases refractory to medical and conservative therapy.

## 1 Introduction

Elephantiasis nostras verrucosa (ENV) is a rare form of chronic lymphedema that causes progressive cutaneous hypertrophy. It can lead to severe disfiguration of body parts with gravity-dependent blood flow, especially the lower extremities [1]. It is characterized by hyperkeratosis and papillomatosis of the epidermis with superimposed hyperkeratotic papulonodules with a verrucose or cobblestone-like appearance [1,2]. Chronic lymphedema, either congenital or secondary to an infection, surgery, radiation, neoplastic obstruction, obesity, portal hypertension, or chronic congestive heart failure, plays an essential role in the pathogenesis of ENV [2]. Management of ENV is often challenging, but a variety of successful medical and surgical treatment strategies have been reported [3].

This work has been reported in accordance with the SCARE criteria [4].

## 2 Case report

A 67 years old male patient presented to the outpatient clinic for 8 years history of multiple scrotal and penile skin lesions and massive non painful scrotal swelling. This swelling was progressive over the years where the patient did not consult any doctor due to this embrassing condition according to him. He denied any recent sexually transmitted disease, genitourinary trauma, and urethral instrumentation. He is a non-smoker and non-alcoholic. He had no sexual intercourse during the last few years. His past medical history was a Gastrointestinal stromal tumor (GIST) of the stomach, stage T1N0M0 diagnosed 3 years ago. He underwent open wedge resection of his GIST tumor and his risk stratification in relation to the location, size and, number of mitosis of the tumor was low. He has been on follow-up for 36 months postoperatively. Serial ultrasonogram and CT scan of the abdomen has been reported to be normal with no evidence of recurrence. Adjuvant imatinib therapy was not considered as the prognostic factors revealed a low risk.

**Fig. 1:**
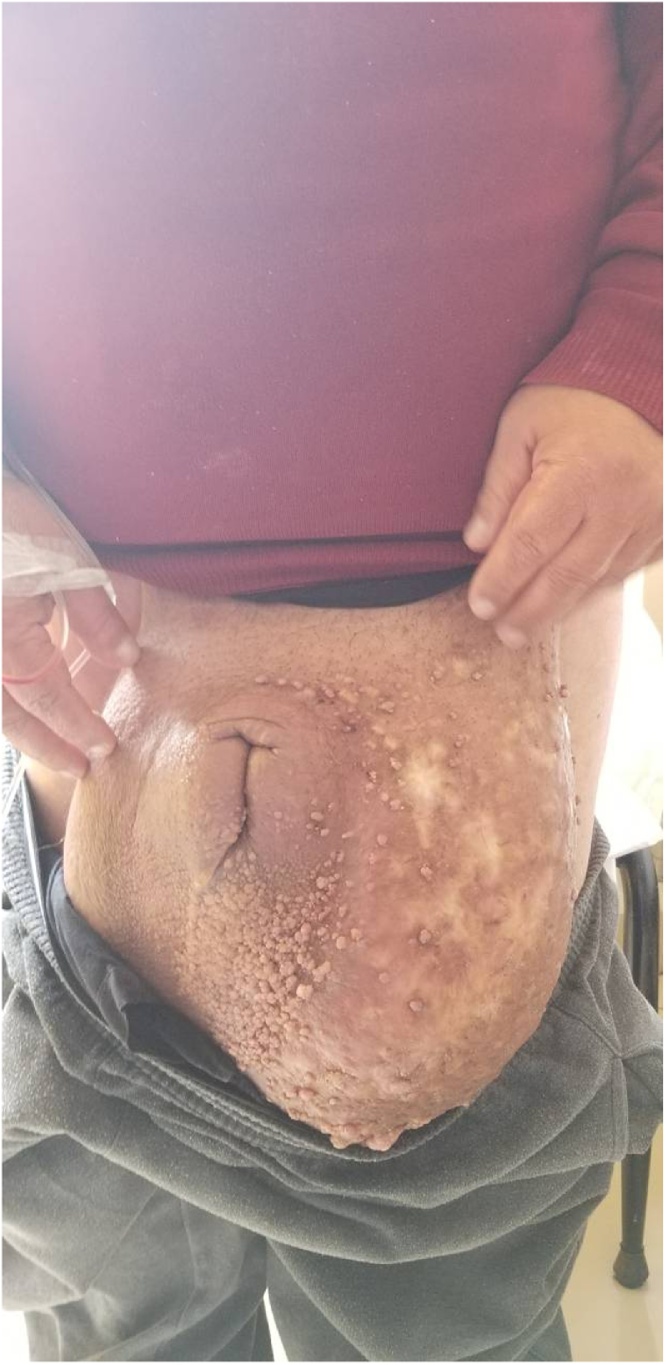
Large swelling of the penis and the scrotum with mossy papules and cobblestone-like nodules.

On admission, his temperature was 37 °C and the vital signs were stable. Physical examination revealed a large swelling of penis and scrotum, generalized thickening of the skin with mossy papules, plaques, and cobblestone-like nodules (Fig. 1). Digital rectal examinations were normal. No dysuria, frequency, hematuria or fever. He has no previous history of sexually transmitted infections or recent travel. He did not have any lymphadenopathy or lower limb edema.

Laboratory examination revealed WBC of 8000/mm [3], Hb of 12 g/dl (normal, 11–16 g/dl). Urine analysis showed 1–2 WBC per high power field. Blood urea & serum creatinine were within the normal limits. HIV test, Serum Venereal Disease Research Laboratory (VDRL), STD panel and Urine culture were negative. Lipid profile together with thyroid, liver, and renal functions were within the normal range. Heart evaluation was normal.

**Fig. 2:**
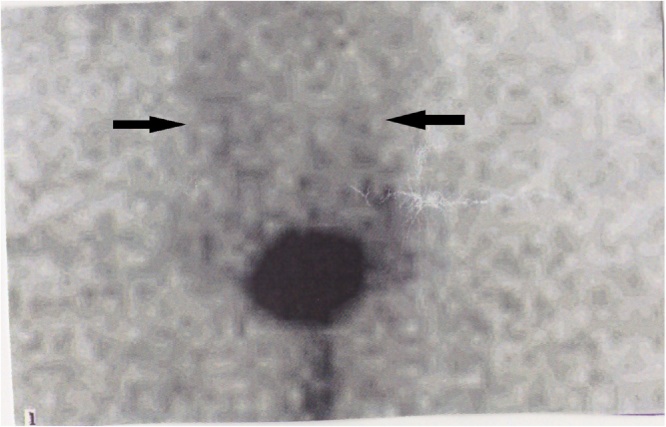
Delayed lymphoscintigraphy image showing no uptake in bilateral inguinal lymph nodes (arrow).

Echography of scrotum revealed extensive soft tissue edema. CT-scan of abdomen and pelvis with and without IV contrast showed no anatomic lesions. Echocardiography was normal with an ejection fraction of 64–68%. Lymphoscintigraphy was performed with one millicurie (mCi) of filtered Technetium sulfur colloid. Multiple intradermal injections were givens in the penis, scrotal sac and perineum. Images showed slow tracer migration bilaterally along inguinal and external iliac lymphatic chains more pronounced on the left side (Fig. 2). Lymphoscintigraphy was consistent with obstruction of inguinal lymphatics. Those findings were consistent with lymphedema.

**Fig. 3:**
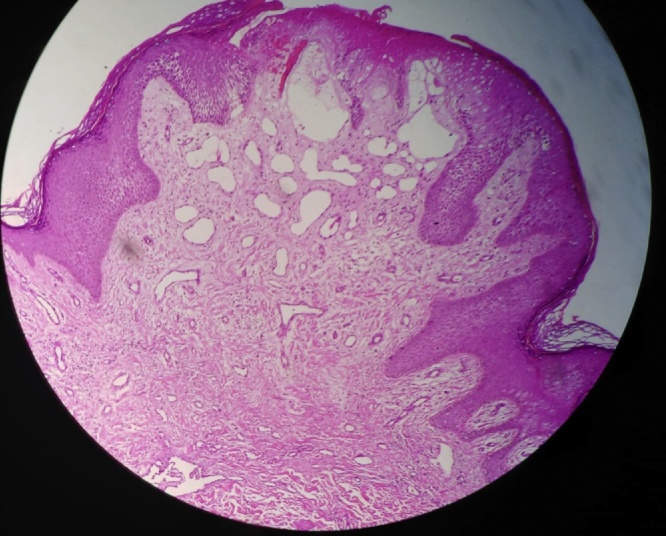
Skin biopsy showing: Hyperkeratosis, irregular epidermal hyperplasia with sparse superficial and midperivascular infiltrate.

Skin biopsy specimens revealed hyperkeratosis, focal epidermal necrosis, a collection of widely dilated vessels in dermis with mild perivascular lymphocytic infiltrate (Fig. 3). These findings were suggestive of lymphedema. The patient was diagnosed with Idiopathic Peno-Scrotal Elephantiasis Nostras Verrucosa (ENV) based on Clinicopathological criteria.

The patient refused surgical excision with full-thickness skin grafting of the penis and scrotum. Conservative options include weight reduction, use of compressive hydrocolloid dressings were given. Oral acitretin 30 mg/day was started to improve cutanenous changes.

During follow up visits, there is a disappearance of some of the hyperkeratotic and verrucous lesion but the treatment was discontinued because of an increase in liver enzymes after 4 weeks. The patient continue to use conservative measures with minimal improvement.

The patient provided a written consent for the publication of this clinical case.

## 3 Discussion

Elephantiasis nostras verrucosa (ENV) is a rare clinical condition associated with chronic non-filarial lymphedema caused by bacterial or non-infectious lymphatic obstruction. In 1934 the term "nostras" was added by Castellani to distinguish the lymphedema of temperate regions not caused by filariasis [5]. The lower extremities are the most common site of ENV, but any area with chronic lymphedema can be affected such as the upper extremities, abdomen, and scrotum [3].

Multiple etiologies can lead to lymphatic obstruction and edema such as bacterial infection, malignancy, lymphangioma, prior surgery or trauma, and lymphatic fibrosis due to radiation therapy, although chronic venous stasis, obesity, and scleroderma should also be considered [1]. Differential diagnoses include chromoblastomycosis, filariasis, lipodermatosclerosis, verrucous carcinoma [3]. Lymphatic filariasis (LF) is a vector-borne disease of the tropical and subtropical countries due to infection by filarial worms, which invade the lymphatics of humans. The nematode species that cause LF include mostly Wuchereria bancrofti to a lesser extent Brugia malayi and Brugia timori. The routine tests like night blood examination to detect microfilariae, Immuno-chromatographic-card test card test for filarial antigenemia [6].

ENV is a serious complication of chronic lymphoedema that causes progressive cutaneous hypertrophy. Lymphoedema, if left untreated, causes fibrosis and sclerosis [3].

History and physical examination are sufficient to diagnose ENV but laboratory tests and other imaging studies may be necessary to exclude other diseases. Lymphatic flow and sites of lymph drainage can readily be evaluated with lymphoscintigraphy. It offers an objective and reliable approach to diagnose and characterize the severity of lymphedema, it also can play an essential role in defining the etiology [7].

Management of ENV is challenging. Therapeutic efforts should aim to reduce lymph stasis which will improve the cutaneous changes [3]. Conservative measures include weight reduction and the use of compression dressing. Surgical excision has been used for some ENV lesions with acceptable results. There are only two single case reports of Peno-Scrotal Elephantiasis Nostras Verrucosa in the literature [8,9]. Judge N et al. described a case of ENV involving the scrotum and perineal area in a 32-year-old man where the lesions were surgically excised, and a full-thickness skin graft was performed, the results were satisfactory [8]. Henshaw EB et al. described the case of a young man with progressively worsening peno-scrotal ENV; highlighting the delay in diagnosis occasioned by the low level awareness of this harrowing affliction among medical practitioners [9].

Systemic retinoids are useful in the treatment of ENV. Zouboulis CC et al. reported the beneficial effect of oral etretinate therapy in an initial dose of 0.6–0.75 mg/kg/day for 4–6 weeks. It induced a rapid disappearance of the hyperkeratotic and verrucous lesions and improvement of lymphoedema [10]. Polat M et al. presented a case of a 64-year-old man with erythrodermic psoriasis and elephantiasis nostras verrucosa in whom the lesions were resolved almost completely after Acitretin treatment [11]. Block VL et al. reported two patients with ENV of the lower limbs which improved with oral Acitretin [12].

In this particular case of isolated penoscrotal ENV, our patient had no history of radiation therapy, trauma, travel to tropical regions, or family history of similar pathology that would have caused his lymphedema. He has only a history of GIST tumor of the stomach that was treated by surgical resection and he had no recurrence during his follow up. The ENV were isolated to the penis and scrotum, it occurs before the diagnosis of GIST by 5 years. Where the correlation between GIST and ENV in such case is not clear, we found the rarity of lymph node metastasis in patients with GIST based on an extensive literature review. Although not a cure and there are side-effects to consider, oral retinoids may offer a significant improvement in ENV as described by multiple reports. In our case oral retinoid showed only a minimal response, this may be due to the short course of therapy where it was discontinued due to its toxicity.

## 4 Conclusion

Management of ENV is usually difficult. It includes oral retinoids and multiple conservative measures. Surgical excision was reported with good cosmetic results when indicated. More data are needed to better define optimal management.

## Declaration of Competing Interest

None identified.

## Sources of funding

No funding.

## Ethical approval

Ethical approval is not required by our institution.

## Consent

Written informed consent was obtained from the patient for publication of this case report and accompanying images.

## Author contribution

Yasmin Moussa, Mohamed Abou Chakra, Mohamad Moussa: Case report design.

Yasmin Moussa, Mohamed Abou Chakra, Mohamad Moussa: Manuscript preparation.

Mohamed Abou Chakra, Mohamad Moussa: Followed up the patient and revised the manuscript.

Yasmin Moussa, Mohamed Abou Chakra, Mohamad Moussa: Approved the final manuscript.

## Registration of research studies

Not applicable, case report.

## Guarantor

Mohamed Abou chakra.

## Provenance and peer review

Not commissioned, externally peer-reviewed.
